# Remission from Kaposi's sarcoma on HAART is associated with suppression of HIV replication and is independent of protease inhibitor therapy

**DOI:** 10.1038/sj.bjc.6603056

**Published:** 2006-03-28

**Authors:** V Martinez, E Caumes, L Gambotti, H Ittah, J-P Morini, J Deleuze, I Gorin, C Katlama, F Bricaire, N Dupin

**Affiliations:** 1 Service de Dermatologie, Hôpital Tarnier-Cochin, AP-HP, UPRES 1833, Université Paris V 89, rue d'Assas, Paris 75006, France; 2 Département des Maladies Infectieuses et Tropicales, Hôpital Pitié-Salpêtrière, AP-HP, Université Pierre et Marie Curie, 45-83, boulevard de l'hôpital, Paris 75013, France; 3 Département de Santé Publique, Hôpital Pitié-Salpêtrière, AP-HP, Université Pierre et Marie Curie, 45-83, boulevard de l'hôpital, Paris 75013, France

**Keywords:** Kaposi's sarcoma, HIV, HAART, naive patients, viral load, CD4 cell counts

## Abstract

Highly active antiretroviral therapy (HAART) reduces the incidence and improves the prognosis of Kaposi's sarcoma (KS). This study was designed to identify factors associated with KS clinical responses in HIV-infected patients during HAART. We reviewed the files of 138 HIV-1-infected patients with KS. Epidemiologic and HIV-related clinical and biological parameters were recorded at KS diagnosis (baseline) and every 6 months thereafter. In a subset of 73 antiretroviral-naive patients, we compared the clinical outcome of KS according to the use or nonuse of protease inhibitors (PI). After 6 months of follow-up, KS remission was more frequent in patients who were naive of HAART and who were at ACTG stage S0 at baseline (*P*=0.03 and 0.02). Undetectable HIV viral load was strongly associated with KS remission (*P*⩽0.004 at all time points), while CD4 cell count was not. Among the 73 antiretroviral-naive patients at baseline, and who were studied for 24 months, KS outcome did not differ between patients who were prescribed PI-containing and PI-sparing regimens. Intercurrent multicentric Castleman's disease was associated with poor outcome after 60 months of follow-up (*P*⩽0.0001). Fourteen deaths occurred after a median follow-up of 37.5 months, eight of which were KS related. Suppression of HIV replication appears to be crucial to control KS. Non-PI-based regimens were equivalent to PI-based regimens as regards the clinical and virological outcome of antiretroviral-naive HIV-infected patients with KS.

Kaposi's sarcoma (KS) has remained the most common AIDS-associated malignancy since the beginning of the HIV/AIDS pandemic (Brodt *et al*, 1997). The introduction of highly active antiretroviral therapy (HAART) in 1996 was associated with undetectable HIV plasma viral load in most patients, together with increased CD4 cell counts, increased survival, and fewer opportunistic infections and AIDS-associated malignancies (Li *et al*, 1998; Palella *et al*, 1998; Levine and Tulpule, 2001). Most studies have shown that KS improves during protease inhibitor (PI)-based antiretroviral treatment, an effect variously attributed to modulation of *Tat* and of production of HIV cytokines, restoration of specific anti-human herpesvirus type 8 (HHV-8) immunity, or the antiangiogenic and antiproliferative effect of PIs (Krischer *et al*, 1998; Lebbe *et al*, 1998; Cattelan *et al*, 1999, 2001; Paparizos *et al*, 2002; Leitch *et al*, 2003). The impact of HAART on immune responses to HHV-8, the main aetiologic agent of KS and multicentric Castleman's disease, is poorly understood. Kaposi's sarcoma lesions are characterised by neoangiogenesis and proliferation of spindle cells of endothelial origin, including cells of lymphatic lineage (Dupin *et al*, 1999). Although experimental studies have shown that PI may have specific antiangiogenic and antiproliferative properties, their specific impact on the clinical outcome of KS has been discussed (Sgadari *et al*, 2002, 2003). Complete remission of KS has been reported in patients receiving PI, and also relapses after switching to NNRTI-based regimens (Murdaca *et al*, 2002; Bani-Sadr *et al*, 2003). In contrast, a significantly lower incidence of KS was observed in HIV-infected patients receiving an NNRTI-based regimen than in those receiving a PI-based regimen in a cohort of 8640 patients (Portsmouth *et al*, 2003). Moreover, NNRTI are also as effective as PIs in prolonging time to treatment failure in KS (Bower *et al*, 1999).

The antiangiogenic action of PI makes these drugs the treatment of choice for HIV-infected patients with KS. However, the so-called PI-sparing regimens are equivalent to PI-based regimens as regards HIV control, while they have better observance and fewer adverse effects (Bucher *et al*, 2003). In order to compare the impact of PI-based and non-PI-based antiretroviral regimens on KS, we reviewed the epidemiological and clinical characteristics and outcome of 138 HIV-1-infected, HAART-treated patients with KS. We also sought factors predictive with KS progression during HAART, analysed the outcome of KS according to CD4 cell count and HIV-1 plasma viral load variations, and compared the benefits of PI and non-PI-based regimens on KS in a subgroup of patients who had not previously received antiretroviral drugs.

## PATIENTS AND METHODS

This retrospective study was conducted in the Department of Infectious Diseases and the Department of Dermatology of two hospitals in Paris, France. Patients were eligible for the study if they had HIV-1 infection and KS, and received HAART before or at the time of KS diagnosis. The time of KS diagnosis was considered as coming on study. Kaposi's sarcoma had to be diagnosed between January 1996 (corresponding to widespread introduction of HAART in France) and May 2001. Protease inhibitor drugs were available in France since January 1996 whereas regimen with NNRTI since 1998. According to their respective availability and international recommandations, triple NRTI or NNRTI-based therapy were thus more likely to be recently prescribed than PI-based therapy. Patients with other neoplasms and those with HHV-8 disease diagnosed before January 1996 were not eligible for the study. The following epidemiological and clinical data were recorded at the time of KS diagnosis (baseline): age, gender, HIV risk group, known duration of HIV infection, the ACTG stage of KS (Krown *et al*, 1997), specific chemotherapy for KS, and associated multicentric Castleman disease. Central, east, west Africa, southern Italy and south America were considered as endemic countries for HHV-8 infection. The occurrence of opportunistic infections or death, and immunologic and virologic parameters of HIV infection (CD4 cell count and HIV-1 plasma viral load) were recorded at baseline and at 6-month intervals throughout follow-up, for outcome of HHV-8-associated malignancies too. HIV plasma viral load was considered undetectable if below 200 copies per millilitre. Specific cytotoxic therapy for KS was used in a part of the patients and included: combination of doxorubicin, daunorubicin, bleomycin, vinblastine, liposomal anthracycline or paclitaxel, monotherapy of bleomycin or vinblastine, IFN-alpha, beta-HCG or local therapy.

In a subgroup of 73 patients who were antiretroviral naive at baseline, we compared the outcome of KS according to the use or non use of PI. Patients were treated and evaluated every 6 months until 24 months under the same combination of antiretroviral drugs. The choice of HAART was left to the patients' individual physicians. ‘KS remission’ refers to partial or complete remission as defined in ACTG study (Krown *et al*, 1997), and ‘KS progression’ refers to stable disease, progressive lesions or death related to KS.

The patient's characteristics were recorded as counts or percentages for categorical variables, and medians and ranges for continuous variables. Categorical variables were compared between patients by using the *χ*^2^ test or Fisher's exact test, as appropriate. Continuous variables were compared using Mann–Whitney test. Differences between groups were considered significant if *P*⩽0.05. A multivariate logistic regression model was used to identify independent predictive factor for KS remission at 6 months by including in the model all the variables which were statistically significant or tended to be in univariate analysis (*P*<0.1). Statistical analysis used the SPSS 11.5 program (Chicago, IL, USA).

## RESULTS

### Baseline characteristics of the patients

We selected and included in this study 138 HIV-1-infected patients from the clinical databases of the two participating units with KS, including five with multicentric Castleman's disease. The baseline characteristics of the 138 patients are shown in Table 1. Median age was 44 years (range 27–73 years). They were 135 men, of whom 101 were homosexual. Kaposi's sarcoma was the first AIDS-defining disease in 43 patients. The 95 other patients were defined as having AIDS according to the CDC classification, before the diagnosis of KS. Thirty-seven patients had already had one or more AIDS-defining events at the onset of KS (nine cytomegalovirus infection, nine atypical mycobacteriosis, eight cerebral toxoplasmosis, five pneumocystosis, six herpes simplex virus infection, three tuberculosis, three non-Hodgkin's lymphoma, two cryptococcosis and one multifocal progressive leukoencephalitis). Forty-one (29.7%) patients originated from a country where HHV-8 is endemic.

At KS diagnosis the median CD4 cell count was 68 mm^−3^ (range 0–797) and the median HIV-1 viral load was 176 000 copies ml^−1^ (range 199–2 831 000) in the whole group. The ACTG stages were predominantly T0, I1 and S1. Sixty-five patients were already receiving HAART and 73 patients had never received antiretroviral therapy when KS was diagnosed but began HAART at this time. In the subgroup of naive patients, the median CD4 cell count was 72 mm^−3^ (range 0–797) and the median HIV-1 viral load was 225 235 copies ml^−1^ (range 3870–2 831 000) whereas in patients already receiving HAART, the corresponding figures were 66 mm^−3^ (range 0–500) and 140 000 copies ml^−1^ (range 199–747 000). Plasma viral load was significantly higher (*P*=0.004) in naive patients than in patients previously treated by HAART whereas CD4 cell counts did not differ (*P*=0.88) between the two groups. For patients already receiving HAART regimen at KS diagnosis, the median duration of HAART was 33 weeks (range 16–98). Highly active antiretroviral therapy consisted of combinations of three NRTI (*n*=17), or two NRTI plus one NNRTI (*n*=12), or two NRTI plus one or two PI (*n*=109) and was chosen by each physician.

### KS treatment and outcome

Seventy-two patients with KS received specific chemotherapy including: adriamycin, bleomycin and vinblastine in 33 cases, paclitaxel in two cases, daunorubicin in two cases, other combinations of drugs in five cases, monotherapy of bleomycin in 17 cases and of vinblastine in six cases, IFN-alpha in three cases, local therapy in three cases and beta-HCG in one case for the first 6 months after diagnosis. Of the 73 naive patients, 40 (55%) underwent a specific chemotherapy and 32 of the 65 (49%) patients previously treated by HAART. The use or not of specific cytotoxic drugs for KS had no impact on the evolution of KS in patients who were already receiving antiretroviral therapy at baseline (*P*=0.32) and in naive patients (*P*=0.59 comparing PI and non-PI-containing regimen). Nevertheless, the use of cytotoxic agents was left at the discretion of the treating physician according to the localisation and extension of the disease. Only five patients were still receiving specific therapy for KS 24 months after diagnosis. The percentage of patients in remission from KS increased during follow-up, from 51% at 6 months to 62% at 12 months, 70% at 18 months and 76% at 24 months. After a median follow-up of 37.5 months (range 6–60 months), 14 patients (10%) had died, of HHV-8 malignancies in eight cases (four with both KS and multicentric Castleman's disease) and of opportunistic infections in six cases.

### Factors predictive of KS outcome in the HAART era

**Figure 1:**
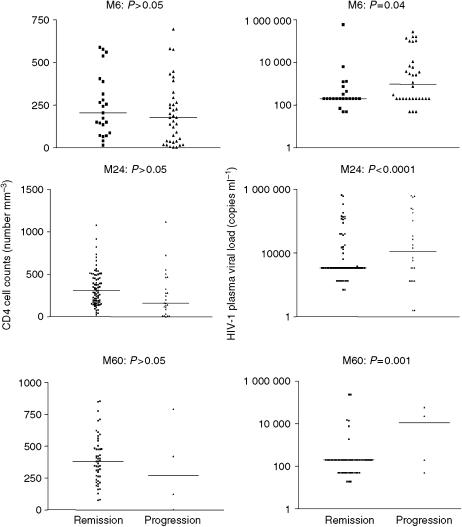
Comparison of HIV plasma viral load and CD4 cell counts between patients with KS remission and progression at months 6, 24 and 60 of follow-up. A logarithmic scale is used for HIV plasma viral load. Horizontal bars represent the medians and many points are at the lower limits of detection of the assay used.

Patients who were antiretroviral naive and those with KS stage S0 at baseline had a significantly higher chance of KS remission during the first 6 months of follow-up (*P*=0.03 and 0.02, respectively) (Table 2). Moreover, in the 73 naive patients, there was a trend to KS remission for patients with stage T0 (*P*=0.08) and stage S0 (*P*=0.07). We also observed a significative trend to remission when AIDS was diagnosed at the time of KS diagnosis (*P*=0.07). Nearly half of the patients developed KS while receiving HAART. This goes counter to the popular wisdom that patients on HAART rarely develop KS. These HAART-experienced patients developed KS at a time when their HIV viral load was poorly controlled (*n*=61) and despite HIV suppression under the level of detection for only four patients. During the first 6 months, KS was in remission in 60.3% of 73 antiretroviral-naive patients, compared to 41.5% of 65 patients who were already receiving antiretroviral therapy at baseline. A highly significant relationship was found between KS remission and undetectable HIV plasma viral load during follow-up (*P*=0.04 at month 6, *P*⩽0.0001 at month 24, *P*=0.001 at months 48 and 60) (Figure 1). This relationship was not observed for CD4 cell count (Figure 1). Multicentric Castleman's disease was associated with KS in five patients, and was associated with poor outcome (*P*⩽0.0001): four patients had died, and one had progressed after 60 months of follow-up. In multivariate analysis only stage S remained significant: a stage S0 patient at baseline has 2.4 (CI 95%: 1.2–5.0) more chance of KS remission during the first 6 months of follow-up than a stage S1 patient. The fact of being antiretroviral naive did not remain significant (*P*=0.07) in the multivariate analysis.

**Figure 2:**
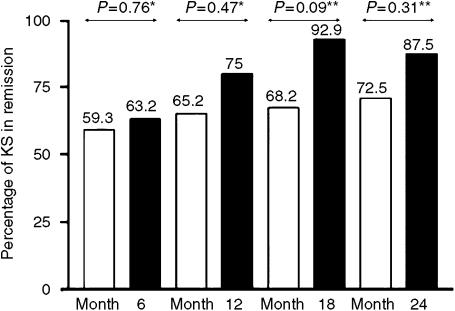
Efficacy of PI-based regimens (white box) and non-PI-based regimens (black box) on KS in 73 HIV-infected antiretroviral-naive patients. Efficacy was evaluated every 6 months for 24 months. ^*^χ^2^ test and ^**^Fisher's exact test.

The 73 patients who were antiretroviral naive at baseline remained on the same HAART regimen for at least 24 months. Fifty-four patients were treated with PI-containing regimens and 19 did not received PI. At baseline, the ACTG stage distribution (*P*=0.46 for stage T, *P*=0.7 for stage S and *P*=0.47 for stage I), CD4 cell counts (median 55 mm^−3^ for non-PI group *vs* 76 mm^−3^ for PI group), and HIV plasma viral load (median 204 000 copies ml^−1^ for non-PI group *vs* 275 000 copies ml^−1^ for PI group) were not statistically different between the two groups (*P*=0.2 and 0.1 for CD4 cell counts and viral load, respectively) and were not significantly related to KS remission at 6 months. During the 24 months of follow-up, the KS remission rate was not significantly different between the two groups (Figure 2): there was no evidence for a better outcome in the patients receiving a PI-containing regimen (Figure 2).

## DISCUSSION

We examined clinical and biological factors associated with KS remission in HIV-1-infected patients receiving HAART. In multivariate analysis, only ACTG KS stage S0 was associated with KS remission at month 6. Intercurrent multicentric Castleman's disease was associated with poor vital outcome and with KS progression. Undetectable HIV-1 plasma viral load was the best predictor of KS remission, independently of the CD4 cell count. In our study, specific chemotherapy in addition to HAART had no impact on KS outcome. Finally, among patients who were antiretroviral naive at baseline, KS outcome was similar in those who received PI- and non-PI-based regimens.

HIV-1 interacts strongly with HHV-8. The HIV *Tat* protein, which is released by infected cells, regulates many viral and host functions. It stimulates the growth of KS lesions by stimulating the proliferation of spindle cells, activating cytokine genes (e.g. *IL-6*) and inhibiting IFN-*γ*-mediated apoptosis (Boshoff *et al*, 1995; Nicholas *et al*, 1997; Deregibus *et al*, 2002). The *Tat* gene has a direct angiogenic effect by interacting with several receptors, and also enhances HHV-8 infectivity (Albini *et al*, 1995; Aoki and Tosato, 2004). Uncontrolled HIV replication is associated with progression of HHV-8 malignancies (Nicholas *et al*, 1997; Brodt *et al*, 1998). As in previous studies, we found that suppression of HIV replication was crucial for controlling KS (Albini *et al*, 1995; Boshoff *et al*, 1995; Nicholas *et al*, 1997; Barillari and Ensoli, 2002; Deregibus *et al*, 2002).

The influence of the CD4 cell count on KS outcome is controversial. Two studies have shown a correlation between KS remission and a high CD4 cell count or a strong increase in the CD4 cell count. Dupont *et al* (2000) found that an increase in the CD4 cell count of more than 150 × 10^6^ l^−1^ after 12 months of HAART was predictive of complete remission from KS at month 24 in HIV-infected patients. Similarly, Cattelan *et al* (1999) found a positive correlation between the CD4 cell count and KS control during antiretroviral therapy. In contrast, like us, Nasti *et al* (2003a, 2003b) found that HIV suppression was associated with good KS outcomes, independently of immune restoration reflected by the CD4 cell count. Undetectable HIV-1 plasma viral load thus appears to be the best predictor of KS remission, independently of the CD4 cell count.

In our subgroup of patients who had not previously received antiretroviral drugs, PI-based regimens and PI-sparing regimens were similarly effective in terms of KS remission. Protease inhibitors can affect important cellular processes such as angiogenesis, tumor growth and invasion, inflammation, antigen processing and presentation, cell survival, tissue remodelling and metastasis (Sgadari *et al*, 2003). Protease inhibitors have direct antiproliferative and antiangiogenic effects *in vitro*, and inhibit the development of KS-like lesions in animal models by blocking an enzyme required for the production of infectious HHV-8 particles (Pati *et al*, 2002; Sgadari *et al*, 2002, 2003). In clinical trials, PI-containing regimens led to full remission from KS in approximately 50% of patients, and conferred an added survival benefit (Burdick *et al*, 1997; Aboulafia, 1998; Krischer *et al*, 1998; Cattelan *et al*, 1999; De Milito *et al*, 1999; Paparizos *et al*, 2002; Leitch *et al*, 2003; Sgadari *et al*, 2003).

In contrast, the impact of NNRTI-based regimens on KS is controversial (Murdaca *et al*, 2002; Bani-Sadr *et al*, 2003; Portsmouth *et al*, 2003). Both complete remissions with NNRTI and relapses of KS in patients switching from PI- to NNRTI-based regimens have been described (Murdaca *et al*, 2002; Bani-Sadr *et al*, 2003). Two prospective studies showed that PI were not essential to clear HHV-8 DNA (Gill *et al*, 2002; Bourboulia *et al*, 2004). Similarly, Stebbing *et al* (2004) showed that NNRTI-based HAART regimens were not inferior to PI-based regimens in preventing KS. NNRTI-containing antiretroviral regimens are as effective as PI-based regimens on HIV infection, but are associated with better adherence, fewer adverse effects, and similar immune restoration (Bucher *et al*, 2003; Pozniak *et al*, 2003). However, PI-containing regimens are superior to NNRTI combinations including nevirapine or delavirdine in patients with advanced immunodepression and previous exposure to NRTI (Yazdanpanah *et al*, 2004). Our results, together with previous reports, suggest that KS regression in this setting is mediated by an overall improvement in immune function, modulation of cytokine expression, and control of HIV and HHV-8 replication, rather than by a direct specific antiangiogenic effect of antiretroviral therapy. It is noteworthy that the effects of NRTI and NNRTI on angiogenesis and proliferation have not been studied. However, we found that PI-sparing regimens were as effective as PI-based regimens in term of KS outcome in antiretroviral-naive HIV-infected patients.

Our patients who were antiretroviral naive at baseline had an increased chance of KS remission during the first 6 months of HAART in the univariate analysis. This might be due to partial recovery of anti-HHV-8 immune function, probable infection by wild-type HIV strains and a presumed better adherence compared to previously HAART-treated patients. Nevertheless, paradoxical transient deterioration have been described in KS patients, as seen in patients with tuberculosis and other opportunistic infections (Narita *et al*, 1998; Baril *et al*, 2000; Stone *et al*, 2002). Shelburne *et al* (2005) showed that patients who have been antiretroviral naive at time of diagnosis of their opportunistic infection have an increased risk of immune inflammatory syndrome. Also, Bourboulia *et al* (2004) reported that anti-HHV-8 immune reconstitution only occurred after more than 24 months of HAART, and that this contributed to the fall in the KS incidence during antiretroviral treatment. Early KS progression (within the first months following HAART introduction) may thus mimic immune reconstitution (Bower *et al*, 2005).

In conclusion, patients with ACTG KS stage S0 at baseline have an increased chance of remission of HHV-8 malignancies during the first six months of HAART. Patients with both KS and multicentric Castleman's disease have a particularly poor prognosis. Suppression of HIV replication by HAART appears to be the key factor in KS control, independently of the CD4 cell count. NNRTI- and NRTI-based regimens appear to be equivalent to PI-based regimens in terms of clinical and virological outcome in antiretroviral-naive HIV-infected patients with KS. It thus seems necessary to define more precisely, in clinical trials, the interest of specific anti-KS drugs on the evolution of KS in HAART era whereas PI- and NNRTI-based regimen seem to have similar efficiency on KS outcome.

## Figures and Tables

**Table 1:** Baseline characteristics of the 138 HIV-infected patients with Kaposi's sarcoma

**Baseline characteristics**	**Number of patients**	**%**
*Gender*		
Male	135	97.8
Female	3	2.2

Multicentric Castleman's disease	5	3.6

Patients from HHV-8-endemic countries	41	29.7

*HIV exposure group*		
Homosexual	101	73.2
Heterosexual	33	23.9
Intravenous drug users	4	2.9

*ACTG TIS classification*		
	79	57.2
T0	59	42.8
T1	37	26.8
I0	101	73.2
I1	49	35.5
S0	89	64.5
S1		

AIDS before KS diagnosis	95	68.8

Opportunistic infections at KS diagnosis	37	26.8

Ongoing HAART at KS diagnosis		
Yes	65	47.1
No	73	52.9


	**Median**	**Range**
Age (years)	44	27–73
CD4 cell counts (per mm^3^)	68	0–797
HIV viral load (copies ml^−1^)^a^	176 000	199–2 831 000

a *n*=101.

**Table 2:** Risk factors for remission at 6 months according to baseline characteristics: univariate and multivariate analysis

	**Remission (*n*=71)**				
	**Univariate analysis**			**Multivariate analysis**	
**Risk factor (no. of patients)**	**No. of patients**	**%**	***P* value^a^**	**OR**	**CI 95%**
*Age (years)*					
⩽44 (71)	34	47.9	0.39		
>44 (67)	37	55.2			

*Sex*					
Men (135)	70	51.9	0.52		
Women (3)	1	33.3			

*HIV exposure group*					
Homosexual (101)	52	51.5	0.37^b^		
Heterosexual (33)	19	57.6			
Intravenous drug user (4)	0	0			

*HHV-8 endemic country of origin*					
Yes (41)	20	48.8	0.68		
No (97)	51	52.6			

*Opportunistic infection at KS diagnosis*					
Yes (37)	17	45.9	0.43		
No (101)	54	53.5			

*AIDS revelated by KS*					
Yes (43)	27	62.8	0.07		
No (95)	44	46.3			

*Antiretroviral naive at baseline*					
Yes (73)	44	60.3	**0.03**		
No (65)	27	41.5			

*T stage*					
T0 (79)	45	57	0.13		
T1 (59)	26	44.1			

*I stage*					
I0 (37)	21	56.8	0.45		
I1 (101)	50	49.5			

*S stage*					
S0 (49)	32	65.3	**0.02**	2.4	[1.2–5.0]
S1 (89)	39	43.8		1 (ref^c^)	

*CD4 cell counts*					
<200 mm^−3^ (100)	50	50	0.58		
⩾200 mm^−3^ (38)	21	55.3			

*HIV-1 plasma viral load*					
⩽200 copies ml^−1^ (5)	3	60	0.83		
>200 copies ml^−1^ (96)	53	55.2			

a Determined with *χ*^2^ Pearson (Fisher's exact test for ‘sex’).

b Without including IDU.

c Reference.
